# Efficient Capture of *Cannabis* Terpenes in Olive Oil during Microwave-Assisted Cannabinoid Decarboxylation †

**DOI:** 10.3390/molecules29040899

**Published:** 2024-02-18

**Authors:** Luisa Boffa, Arianna Binello, Giancarlo Cravotto

**Affiliations:** Dipartimento di Scienza e Tecnologia del Farmaco, University of Turin, Via P. Giuria 9, 10125 Turin, Italy; luisa.boffa@unito.it (L.B.); arianna.binello@unito.it (A.B.)

**Keywords:** *Cannabis* inflorescences, selective extraction, microwave-assisted decarboxylation, terpene capture, flavored olive oil, HS-SPME GC-MS analysis

## Abstract

The development of selective extraction protocols for *Cannabis*-inflorescence constituents is still a significant challenge. The characteristic *Cannabis* fragrance can be mainly ascribed to monoterpenes, sesquiterpenes and oxygenated terpenoids. This work investigates the entrapment of *Cannabis* terpenes in olive oil from inflorescences via stripping under mild vacuum during the rapid microwave-assisted decarboxylation of cannabinoids (MW, 120 °C, 30 min) and after subsequent extraction of cannabinoids (60 and 100 °C). The profiles of the volatiles collected in the oil samples before and after the extraction step were evaluated using static headspace solid-phase microextraction (HS-SPME), followed by gas chromatography coupled to mass spectrometry (GC-MS). Between the three fractions obtained, the first shows the highest volatile content (~37,400 mg/kg oil), with α-pinene, β-pinene, β-myrcene, limonene and *trans*-β-caryophyllene as the main components. The MW-assisted extraction at 60 and 100 °C of inflorescences using the collected oil fractions allowed an increase of 70% and 86% of total terpene content, respectively. Considering the initial terpene amount of 91,324.7 ± 2774.4 mg/kg dry inflorescences, the percentage of recovery after decarboxylation was close to 58% (mainly monoterpenes), while it reached nearly 100% (including sesquiterpenes) after extraction. The selective and efficient extraction of volatile compounds, while avoiding direct contact between the matrix and extraction solvents, paves the way for specific applications in various aromatic plants. In this context, aromatized extracts can be employed to create innovative Cannabis-based products within the hemp processing industry, as well as in perfumery, cosmetics, dietary supplements, food, and the pharmaceutical industry.

## 1. Introduction

As medical *Cannabis* is legally available for patients in several countries, it has become extremely important to develop fast, accurate and efficient analytical protocols to determine not only the contaminants and main bioactive substances, but also terpene identity and concentration levels in different strains [1].

The therapeutic effects of *Cannabis* certainly depend on its phytocannabinoid content and profile, with the most relevant being Δ^9^-tetrahydrocannabinolic acid (THCA) and cannabidiolic acid (CBDA) [2]. In order to obtain their neutral homologues THC and CBD, which display high affinities for cannabimimetic activity receptor (CARe), they are usually decarboxylated via heating during consumption or during extraction processes [3,4]. The decarboxylation step consists of heating treatment carried out at a temperature above 100 °C, but below 230 °C, to avoid the formation of smoke toxins [5,6].

The *Cannabis sativa* L. phytocomplex includes a significant number of substances beyond cannabinoids that belong to the terpene, fatty acid, alkaloid, carbohydrate and polyphenol families. Similar to cannabinoids, more than 100 terpenes and terpenoids have been identified and characterized in the *Cannabis* plant, and are usually present in consumer products [7,8]. Common terpenes found in *Cannabis* include monoterpenes, such as limonene, α- and β-pinene, β-myrcene and terpinolene, and sesquiterpenes, such as α-humulene and β-caryophyllene. A number of terpenoids, which contain other elements such as oxygen, can also be easily observed, including α-fenchyl alcohol, α-terpineol, linalool, borneol, guaiol, α-bisabolol, and caryophyllene oxide [8]. The terpene and terpenoid profiles can be associated with the geographical origin of the *Cannabis* plant or product [9,10] and to specific strains, which have been developed with distinct aromas and flavors [11]. It has been reported that some terpenes possess antimicrobial, anti-inflammatory, immuno-modulatory, anticancer and anti-anxiety properties [12,13,14,15], but they are best recognized for their flavor and aroma, which contribute to consumer preferences (e.g., essential oils and food flavoring) [16]. This explains the choice of *Cannabis* for recreational use by consumers and the possible synergistic effects of cannabinoids and terpenes with regard to medical benefits [17,18,19].

Hence, the comprehensive and accurate qualitative and quantitative analysis of this class of natural compounds, together with cannabinoids, is essential for guaranteeing the quality of *Cannabis* used in medical treatment.

The typical analytical protocol for terpenes in plant materials involves an extraction step, such as conventional maceration, steam and hydro-distillation [20,21,22] and supercritical-CO_2_ [23], followed by gas chromatography (GC) analysis. The combination of mass spectrometry (MS) and chromatographic separation can considerably improve the efficiency of the analytical method [24]. The use of automated sample preparation methods, such as static headspace (SHS) [8,25] and solid-phase microextraction (SPME) [25,26], means that the extraction step can be avoided and can reduce or eliminate sample incompatibility with the analytical systems, limit matrix interference, while also, more importantly, concentrating the samples [27].

A few papers have reported the SHS/GC-MS analyses of *Cannabis* terpenoids. If the injection of HS does not reach adequate sensitivity, analytes can be directly extracted and concentrated onto sorbent polymers, which may improve detection limits, while integrating sampling, extraction, concentration and sample introduction into a single solvent-free step. HS-SPME is an emerging analytical extraction technique for the sample preparation of *Cannabis*-based products, and several papers [25,28,29] and technical reports [26,30,31,32,33,34] have described SPME as an extraction method for the analysis of this plant.

A study on different SPME fibers (CAR-PDMS, PDMS, PDMS-DVB or divinylbenzene, CAR-PDMS-DVB) demonstrated that DVB/CAR/PDMS was able to extract a higher amount of terpenes than the others [29], confirmed also by other reports [31,32,34]. In a recent work, Giovannoni et al. reported that extraction efficiencies were higher when using 50/30 μm DVB/CAR/PDMS fibers, especially for more volatile compounds (monoterpenes), but the 30 μm PDMS fiber allowed them to obtain larger peak heights for sesquiterpenes, avoiding the saturation of monoterpene signals [25].

Lastly, a method using the capillary microextraction of volatiles (CMV), which is based on planar solid-phase microextraction (PSPME), has been reported by Wiebelhaus [35]. The technique was found to be capable of collecting trace amounts of volatile compounds in a short extraction time (1 min), enabling the development of a technique for field testing the presence of marijuana plants.

To date, several commercial *Cannabis*-based products have been approved for medical use (e.g., Cesamet, Epidiolex, Marinol, and Sativex) [36,37,38]. However, many galenic formulas can be prepared by pharmacists in accordance with medical prescriptions, with this process involving the extraction of dried female inflorescences in olive oil or ethanol, with or without the previous decarboxylation of acidic cannabinoids [29,39]. Phytocannabinoids can be exhaustively extracted from dried inflorescences with ethanol [40], but the use of vegetable oils, such as olive oil, has been proposed as a more environmentally friendly extraction method.

In 2013, Romano and Hazekamp published one of the first works on the chemical characterization of *Cannabis*–oil preparations, which were obtained via decarboxylation and extraction in a conventional heated water bath [41], finding evidence that the decarboxylation step led to significant losses in terpene components. Compared to the untreated control, monoterpenes (the most volatile) were reduced to about half of their original levels even after 5 min of exposure to boiling water, while only small traces could still be detected after the more intense oven treatment. Subsequently, Citti et al. [39] proposed a simple method for the preservation of terpenes in *Cannabis*-based medicinal extracts, introducing a reflux system in which the inflorescences were extracted with either ethanol or the more sustainable olive oil, and also observed the greatest terpene recovery in the more environmentally friendly solvent.

In 2018, Calvi et al. [29] performed a decarboxylation step of hemp inflorescences in a static oven at 145 °C for 30 min, and then the extraction of cannabinoids under US (35 KHz, 30 min). The maximum volatiles content was found in the control oil, which was produced without exposing the *Cannabis* inflorescences to the decarboxylation step.

A high extraction efficiency for terpenes was also provided by two alternative methods in which the essential oil obtained from the steam distillation or microwave-assisted hydrodistillation (MAHD) (ETHOS X apparatus, Milestone Srl, Sorisole, BG, Italy) of plant inflorescences was then added to the oil extracted from the residual plant by dynamic maceration at room temperature [34].

Our most recent paper [42] investigated a new prototype reactor for the fast decarboxylation of acidic cannabinoids under microwaves (MW) and their subsequent extraction into olive oil. The decarboxylating step was kept as short as possible (30 min, 120 °C) to decrease the loss of volatiles from *Cannabis* inflorescences.

Based on these promising extraction results and considering the importance of terpenes for their synergistic effects with phytocannabinoids, the present work proposes to improve the same MW-assisted protocol carried out in the MW Ethos LEAN oven (Milestone Srl) to try to capture all the volatiles of decarboxylation in different fractions of olive oil, which are subsequently diluted and used as solvent for the extraction of cannabinoids.

Firstly, the absolute and percentage composition of terpenes and sesquiterpenes in the inflorescences was evaluated. The values obtained served as a reference for comparison with the volatile compositions of both the terpene-enriched oil fractions and the products obtained by MW-assisted extraction (MAE) (at 60 and 100 °C) of the decarboxylated inflorescences, in order to understand the effectiveness of the protocol in capturing all the volatiles present in the original inflorescences. Analyses were performed by pre-concentration of the volatiles via HS-SPME followed by identification and semi-quantification by GC-MS.

## 2. Results and Discussion

**Figure 1:**
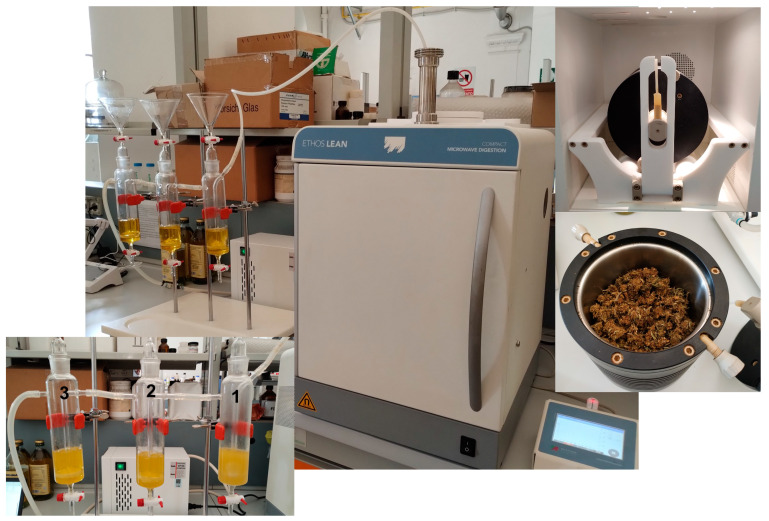
Ethos LEAN MW oven configuration for the capture of *Cannabis* terpenes in olive oil (Milestone Srl).

The first step was the accurate configuration of the MW prototype reactor (Ethos LEAN, Milestone; Figure 1). A dedicated vessel (rotating drum; see Figure 1 on the right) was filled with dried *Cannabis* inflorescences, closed and placed inside the oven cavity. The vessel output was connected to three sequential glass bubblers (identified as 1, 2 and 3 from the closest to the MW oven) through PTFE tubes with sealed connections, which were placed outside the oven and connected to a mild vacuum to help air flow from the reactor (Figure 1 on the left). Each bubbler was filled with olive oil (100 mL, Ph. Eur. grade). The fast MW-assisted decarboxylation of acidic cannabinoids was carried out at 120 °C (30 min) with vessel rotation, as described by Binello et al. [42]. During this process, the volatiles coming from the rotating drum were trapped in the oil fractions (F1, F2 and F3, and no odor was perceived outside the MW cavity. After a cooling step, the drum was removed from the oven and the plant matrix was recovered and used for cannabinoid extraction, while the three oil fractions were mixed and diluted to 1 L with fresh olive oil (Mix F1 ÷ 3 dil.). Decarboxylated inflorescences were placed in a glass reactor together with the prepared olive oil (matrix/solvent ratio 1:10) and heated under MW irradiation with stirring at 100 °C for either 30 min (Ext. 100) or at 60 °C for 30 min using a cold finger, maintained at 8 °C and directly inserted into the oil (Ext. 60). At the end of the final cooling step, the oil samples were filtered under vacuum to recover the extract in oil. For all samples (F1, F2, F3, Mix F1 ÷ 3 dil., Ext. 60 and Ext. 100), an aliquot of 10 mL was taken for analysis.

**Figure 2:**
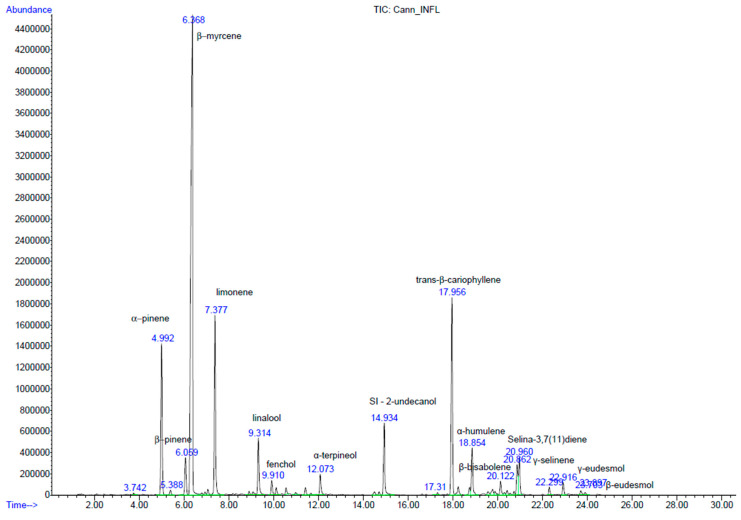
Chromatogram obtained from the SPME/GC-MS of *Cannabis* inflorescences with 2-undecanol as IS (14.93 min).

As outlined in the first paragraph, HS-SPME has been considered the analytical extraction technique of choice for *Cannabis*-based preparations and products in recent years [25,28,29]. Our protocol is based on Merck technical reports [31,32]. A DVB/CAR/PDMS fiber assembly (1 cm, 50/30 µm film thickness, Supelco, Bellefonte, PA, USA) was used with the same injector and detector temperatures, oven ramp and mass parameters being applied (see Section 3). However, a Mega-5 MS column, as in Citti et al. [39], an equilibration time of 10 min and an extraction time of 40 min were used as SPME parameters, as in the Aparicio-Ruiz method for the analysis of volatile compounds in olive oils [43]. The preparation of the samples and internal standard solutions (IS) was also carried out following the detailed protocol in this article, using 2-undecanol as the IS (2.0 ± 0.2 mg/g in olive oil) and by adding 0.2 g of IS solution to 2.8 g of the aromatic oil samples. The inflorescences were ground in a laboratory blender under liquid nitrogen, and samples were then prepared for SPME analysis by adding 0.2 g of IS solution to 100 mg of plant material. Details are reported in the Section 3. A blank with the olive oil used for trapping the volatiles and cannabinoid extraction was prepared and analyzed under the same conditions to verify that its oil aromatic profile did not interfere with the retention times of the IS (14.93 min, Figure 2) and *Cannabis* terpenes (between 4 and 9 min for monoterpenes, 9 and 12 min for monoterpenoids, 17 and 21 min for sesquiterpenes and 22 and 24 min for sesquiterpenoids; Figure 2 and Table 1).

The inflorescences showed a total amount of terpenes and terpenoids equal to 91,324.74 ± 2774.36 mg/kg dry matrix (Table 1), which is quite similar to the value found in Bedrocan^®^ (~111,400 mg/kg), while higher than that observed in Bediol^®^ (~64,000 mg/kg) [29]. Of the 28 identified, the main compounds were α- and β-pinene (9 and 2.5%), β-myrcene (44.3%), limonene (10.6%), linalool (3.5%), d-α-Fenchyl alcohol (0.9%), α-terpineol (1.4%), *trans*-β-caryophyllene (12.8%), α-humulene (3.3%), β-bisabolene (0.9%), γ-selinene (1.9%), selina-3,7(11)diene (2.6%) and γ-eudesmol (1%), as indicated in Table 1 and shown in Figure 2.

**Figure 3:**
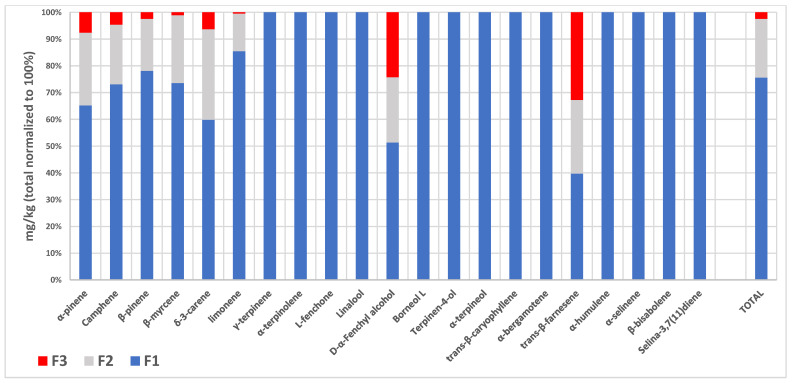
Hundred-percent stacked column representation of the average ppm data from Table 2, obtained from SPME/GC-MS analyses.

**Figure 4:**
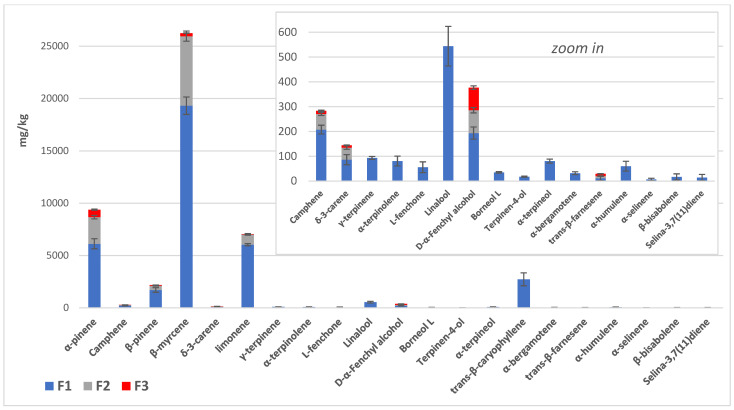
Stacked column representation of the terpene composition of the three oil fractions (F1, F2 and F3) (expressed as mg/kg oil, average ± S.D., data in Table 2), obtained from SPME/GC-MS analyses.

The oil fractions in the bubblers (F1, F2, F3, see Table 2 and Figure 3 and Figure 4) were analyzed by SPME/GC-MS after the decarboxylation of *Cannabis* inflorescences. F1 showed the highest volatile content (37,390.38 ± 723.16 mg/kg oil), with α-pinene, β-pinene, β-myrcene, limonene and *trans*-β-caryophyllene being the most important of the 21 components identified. F2 and F3 showed terpene contents of 10,819.59 ± 187.83 and 1208.78 ± 31.12 mg/kg oil, respectively, with only eight compounds detectable. In the hundred-percent stacked column representation of the average ppm data from Table 2 (Figure 3), it can be observed that over 92% of the single terpenes (with fenchyl alcohol and *trans*-β-farnesene being excluded) were trapped in the first two oil fractions; this increased to 98% when the total amount was considered. Figure 4, which is a stacked column representation of the terpene composition of the three oil fractions (F1, F2 and F3), highlights the main compounds present and their different abundances in each oil fraction, confirming that the first two are the richest in volatiles.

**Figure 5:**
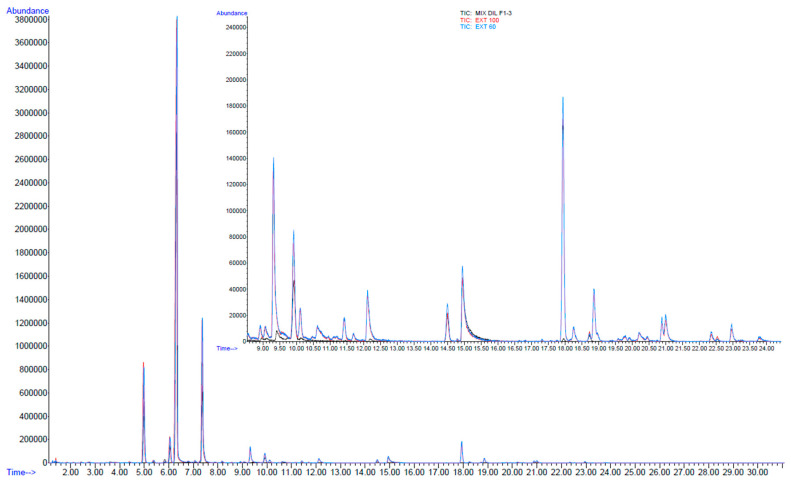
Overlapped chromatograms obtained from the SPME/GC-MS of mixed and diluted (Mix F1 ÷ 3 dil.) oil fractions and oil samples from MAE at 60 °C and 100 °C (Ext. 60, Ext. 100).

**Figure 6:**
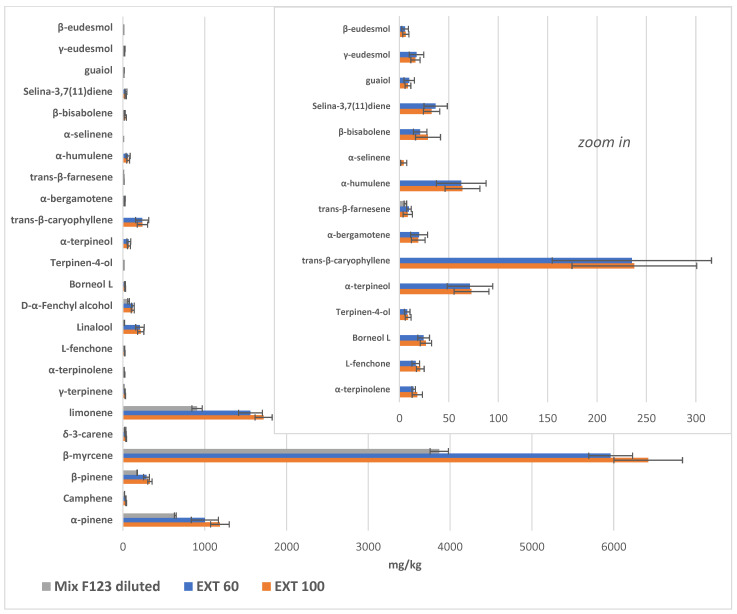
Parallel column representation of the terpene composition of oil obtained after extraction at 60 and 100 °C, compared to the starting sample (Mix F1 ÷ 3 dil.), expressed as mg/kg oil (mean ± standard deviation). Data were obtained from SPME/GC-MS analyses and are shown in Table 3.

The oil obtained from a 1:1:1 mixture of F1, F2 and F3 and its dilution to 1 L in total (Mix. F1 ÷ 3 dil., Table 3) displayed 5733.97 ± 167.35 mg/kg of terpenes, while 9775.56 ± 678.04 and 10,672.39 ± 723.05 mg/kg are the total terpene contents of the oils obtained from MAE at 60 and 100 °C, respectively, showing a significant difference from initial sample (Ext. 60 and Ext. 100, Tukey’s post hoc test groups for Totals in Table 3). While Mix F1 ÷ 3 dil. was observed to predominantly contain monoterpenes and monoterpenoids (Figure 5 and Figure 6), the olive oils obtained from the MAE of decarboxylated inflorescences indicated the presence of different compounds, especially sesquiterpenes and sesquiterpenoids (sign + in Table 3 and zoom of Figure 5 and Figure 6 on the right).

All the other compounds were present in amounts that were higher by between 15 (δ-3-carene) and 1100% (linalool) compared to the starting olive oil (Mix F1 ÷ 3 dil.), with an overall increase of 70% and 86% in the total amount of terpenes found in the oils (Table 3), for Ext. 60 and Ext. 100, respectively. Although the trend seems to indicate that extraction at 100 °C gave a higher amount of terpenes than extraction at 60 °C, the differences were not statistically significant (see Tukey’s post hoc test groups for Totals in Table 3).

The highest terpene total amounts in oils obtained from Bedrocan^®^ and Bediol^®^ by Calvi et al. [29] were around 3700 and 1900 mg/kg, respectively, when *Cannabis* inflorescences were not exposed to the decarboxylation step before extraction. Around 1500 and 900 mg/kg, respectively, were obtained using the Romano and Hazekamp method after decarboxylation [41], demonstrating the significant loss of terpene components. Considering that a total terpene amount of 111,424 mg/kg dry matrix is present in Bedrocan^®^, the oil obtained (1 g inflorescences/10 mL of olive oil; d = 0.92 g/mL) without decarboxylation (3700 mg/kg oil) showed a terpene recovery of only 34%. As reported in Table 4, if we consider that the initial content of terpenes in inflorescences is 91,324.74 ± 2774.36 mg/kg dry matrix, the recovery percentage was close to 58% after decarboxylation (predominantly monoterpenes), while it reached 98.48 ± 6.83% and 107.51 ± 7.28% after extraction at 60 and 100 °C (including sesquiterpenes). Ternelli et al., have recently proposed several ways to obtain an oil that is rich in terpenes, including the use of milder decarboxylation (115 °C for prolonged time) in a closed flask before the extraction, and the addition of the essential oil obtained from steam distillation or MW-assisted hydro-distillation of plant inflorescences to the olive oil subsequently prepared by extracting the residual plant matrix via dynamic maceration at room temperature [34]. However, the article did not report the amount of terpenes (mg/kg) obtained in the oils using the different methods, nor the *w*/*w* % recovery of terpenes from the starting inflorescences. 

**Figure 7:**
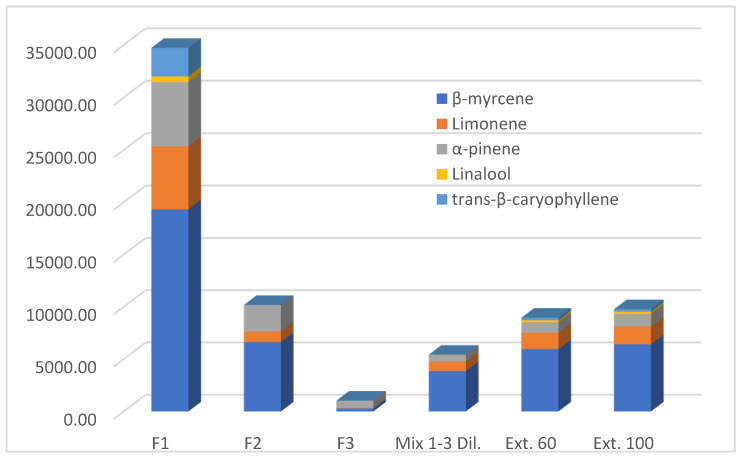
Comparison of the main terpene amounts (mg/kg oil) present in the oily samples obtained during decarboxylation (F1, F2 and F3) and after extraction of decarboxylated inflorescences at 60 and 100 °C (Ext. 60 and Ext. 100), using Mix F1 ÷ 3 dil. as solvent.

Finally, Figure 7 shows the trend in the amount of the main terpenes in our samples, whose principal bioactivity (e.g., anti-inflammatory, sedative, analgesic, antimicrobial, anticancer, or antioxidant) is reported in the literature [12]. Considering the fact that in the oil used as a solvent, the volatile components collected during decarboxylation undergo a dilution step, the final inflorescence extracts showed a comparable relative distribution, with the influence of F1 clearly predominant.

## 3. Materials and Methods

### 3.1. Plant Material

Dried inflorescences (moisture near 5%) of *Cannabis sativa* L. (8.1% CBD and 0.18% THC) were obtained in sealed plastic bags under vacuum from Società Agricola F.lli Podimani S.S. via Avv. G. Cartia 356, Ragusa. The inflorescences were crushed before the decarboxylation step and extraction with olive oil.

### 3.2. Chemical Reagents and Solvents

The standards (*Cannabis* Terpenes Mix A, Cannabis Terpenes Mix B) and 2-undecanol were purchased from Sigma-Aldrich (St. Louis, MO, USA). Ph. Eur. grade olive oil was purchased from a pharmacy.

### 3.3. Extraction Protocol

The decarboxylation and extraction processes were carried out in an Ethos LEAN (Milestone Srl, Sorisole, BG, Italy) professional MW oven, equipped with a 900 W magnetron and an infrared pyrometer for temperature measurement. Integrated software (*Easy Control* by Milestone) allows the setting and control of process parameters such as temperature, time and power.

#### 3.3.1. Decarboxylation in the Milestone Ethos LEAN Prototype and Volatile Trapping

Following the published optimized protocol [42], a given amount of chopped vegetable matrix (100 g) was placed in the drum located within the cavity of the MW oven and then heated at 120 °C under rotation (15 min from r.t. to 120 °C, 900 W max; 30 min at 120 °C, 900 W max; 10 min cooling step). Three bubblers were connected at the end of the reactor tube and were each filled with 100 mL of olive oil. After cooling, the drum was removed from the oven cavity and the plant matrix was recovered and used for further transformations. F1, F2 and F3 were combined and brought to the volume of 1 L with olive oil, giving the solvent for the subsequent MAE extraction of cannabinoids (Mix F1 ÷ 3 dil.) from the residual inflorescences. Three different aliquots of each sample (F1, F2, F3 and Mix F1 ÷ 3 dil.) were collected for triplicate analysis.

#### 3.3.2. Extraction in Olive Oil Using the Milestone Ethos Lean Prototype

A given amount of plant matrix (~50 g) was placed in the glass reactor (2 L) together with Ph. Eur. (European Pharmacopoeia) olive oil (matrix/solvent ratio 1:10, 500 mL) and heated under MW irradiation (2.45 GHz), with stirring, either at 100 °C (10 min from r.t. to 100 °C, 900 W max; 30 min at 100 °C, 900 W max; 20 min cooling step) or 60 °C using a cold finger, refrigerated at 8 °C, and directly inserted into the oil (10 min from r.t. to 60 °C, 900 W max; 30 min at 60 °C, 900 W max; and 20 min cooling step). The two different temperatures were tested to evaluate their possible effects on the final terpene amount. At the end of the cooling step, oil samples were filtered under vacuum to recover the extract in oil. Three different aliquots of obtained samples (Ext. 60 and Ext. 100) were collected for triplicate analysis.

### 3.4. HS-SPME GC-MS Analyses of Oil and Inflorescence Samples

#### 3.4.1. Equipment

GC analyses were performed using an Agilent 6850 gas chromatograph oven equipped with a split/splitless injector and a SPME injector liner (0.75 mm ID) and coupled with an Agilent 5973N Mass Selective Detector (MS). The installed capillary column was a Mega-5MS 5% Phenyl Methyl (length 30.0 m, ID 0.25 mm, film thickness: 0.25 µm, MEGA S.r.l., Legnano, Italy).

For SPME analyses, a Supelco DVB/CAR/PDMS fiber assembly, length 1 cm, 50/30 µm film thickness (fused silica 24 Ga, gray) was installed in a manual holder and then used for the extraction of volatiles from samples, which were weighed directly in headspace glass vials (20 mL) and hermetically sealed before equilibration.

#### 3.4.2. Instrument Parameters

Oven temperature ramp: initial temperature 60 °C for 2.00 min, ramp of 5.00 °C/min up to 275 °C, held for 5.00 min. Inlet: split mode with a 5:1 split ratio; temperature: 270 °C; gas carrier: helium at a constant flow of 1.2 mL/min. Average velocity: 40 cm/sec. Mass detector: temperature of 300 °C, MS Source 230 °C, MS Quad 150 °C. Acquisition mode: Scan. Resulting EM Voltage: 1612. Mass range: 50–500.

#### 3.4.3. Sampling Mode

DVB/CAR/PDMS fibers were exposed at the inlet port of the GC oven at 250 °C for 2–4 cleaning cycles before use. Samples were equilibrated for 10 min at 40 °C under magnetic stirring (250 rpm), and the SPME fiber was then exposed in the headspace of the sample for 40 min at 40 °C under magnetic stirring (250 rpm). Finally, the fibers were desorbed at the inlet port of the GC oven at 270 °C for 5 min (sample injection).

#### 3.4.4. Sample Preparation

Following the well-detailed method recently published by Aparicio-Ruiz et al. [43], an internal standard solution was prepared by weighing 2-undecanol (around 5 mg, IS) in a vial and then adding the olive oil used for terpene trapping (around 2.8 g). The concentration of the final solution was 2.0 ± 0.2 mg/g of IS in olive oil. An oil blank was prepared and analyzed under the same conditions to verify that the oil aromatic profile did not interfere with the IS and the cannabis terpenes. The oil samples were prepared by adding 0.21 ± 0.02 g of IS solution to 2.8 ± 0.02 g of the aromatic oils (Fraction 1, 2 and 3, Mix F1 ÷ 3 Dil., Ext. 60, Ext. 100) in a headspace vial to achieve an 2-undecanol amount of between 400 and 450 µg. The inflorescence samples were prepared by adding 0.21 ± 0.02 g of IS solution to 100 ± 0.2 mg of ground plant material (in a lab blender under liquid nitrogen). Analyses were performed in triplicate and expressed as means ± standard deviation (S.D.).

#### 3.4.5. Identification and Quantification

Compounds were considered to be positively identified when electron-impact (EI) mass spectra matched with Wiley7n and NIST11 libraries with a minimum quality of 90%. The identification of α-pinene, camphene, β-pinene, δ-3-carene, limonene, γ-terpinene, α-terpinolene, l-fenchone, linalool, d-α-fenchyl alcohol, borneol L, α-terpineol, *trans*-β-caryophyllene, α-humulene and β-eudesmol was performed based on standard retention times (*Cannabis* terpene mix A and B, Certified reference Material from Sigma-Aldrich), while β-myrcene, terpinen-4-ol, α-bergamotene, *trans*-β-farnesene, valencene, α-selinene, β-bisabolene, α-gurjunene, γ-selinene, selina-3,7(11)diene, guaiol, γ-eudesmol and (+)-bulnesol were identified by library matching.

The semi-quantitative analysis of flavor compounds (FC) in hop samples was performed based on the 2-undecanol amount (mg), used as an IS, using the following formula:

µg FC = µg IS × Area FC/Area IS

### 3.5. Statistical Analysis

The statistical analyses were performed using R software (R Foundation for Statis-tical Computing, Vienna, Austria), version 3.6.3 (29 February 2020). The analyses were all performed in triplicate. The residuals were checked for normality using the Shapiro–Wilk test and the Kolmogorov–Smirnov test and the variances were tested for homogeneity using the Levene’s test. The data in Table 3 were processed by single-way ANOVA statistical techniques. Tukey’s HSD post hoc multiple comparison test was performed (confidence level of 0.95, significance level = 0.05) to determine the differences between the different sample means.

## 4. Conclusions

This work presents an efficient and selective method for the capture of terpenes from *Cannabis* inflorescences in olive oil during the MW-assisted decarboxylation of acidic cannabinoids. The obtained perfumed oily solution has been used as a solvent for the rapid MW-assisted extraction of cannabinoids at different temperatures (60 and 100 °C). In view of the initial amount of terpenes in the inflorescences obtained by SPME analyses, the percentage of their recovery after decarboxylation was close to 50%, reaching nearly 100% after extraction. Unlike hydrodistillation, the cannabinoids are not dragged into the glass bubblers containing the olive oil.

The selective and efficient extraction of volatile compounds, avoiding direct contact between the matrix and extraction solvents, opens the door for specific application for several aromatic plants, thus averting the risk of contamination. This terpenes enriched oil can be used to flavor food and beverages and as a solvent in *Cannabis*-based medical formulations. In the latter case, the synergistic effects between terpenes and cannabinoids could be maximized in the final products, with enhanced therapeutic properties. Finally, the method’s versatility in trapping terpenes and other volatile fractions from different matrices suggest the possibility to extend the protocol to other green solvents, as glycerol, vegetal oils or deep eutectic solvents.

## Figures and Tables

**Table 1:** Terpenes present in *Cannabis* inflorescences, expressed as mg/kg dry matrix (mean ± standard deviation) and in area percentages over the total area of listed compounds (mean ± standard deviation), obtained from SPME/GC-MS analyses.

Compounds	Ret. Time (min) ^a^	Ident. (ST/Qual. %) ^b^	Inflorescences (mg/kg, Mean ± S.D.) ^c^	Area Percentage (%, Mean ± S.D.) ^c^
*Monoterpenes*				
α-pinene	5.00	CTA	8265.21 ± 624.95	9.04 ± 0.43
Camphene	5.40	CTA	294.56 ± 20.49	0.32 ± 0.02
β-pinene	6.06	CTA/B	2316.61 ± 139.99	2.54 ± 0.12
β-myrcene	6.30	97	40,459.89 ± 1402.91	44.34 ± 2.24
δ-3-carene	6.79	CTA/B	175.21 ± 17.47	0.19 ± 0.02
limonene	7.40	CTA/B	9660.13 ± 381.68	10.59 ± 0.57
γ-terpinene	8.17	CTA	16.57 ± 33.14	0.02 ± 0.04
α-terpinolene	8.90	CTB	175.11 ± 7.68	0.19 ± 0.01
*Monoterpenoids*				
l-fenchone	9.08	CTA	229.05 ± 28.78	0.25 ± 0.03
Linalool	9.30	CTB	3228.18 ± 154.61	3.53 ± 0.09
d-α-Fenchyl alcohol	9.90	CTA	809.58 ± 99.82	0.89 ± 0.09
Borneol L	11.43	CTB	406.24 ± 23.73	0.44 ± 0.02
Terpinen-4-ol	11.67	90	74.89 ± 50.98	0.08 ± 0.06
α-terpineol	12.10	CTB	1289.09 ± 60.39	1.41 ± 0.04
*Sesquiterpenes*				
*trans*-β-caryophyllene	17.90	CTB	11,696.54 ± 999.88	12.80 ± 0.79
α-bergamotene	18.20	94	656.90 ± 63.10	0.72 ± 0.06
*trans*-β-farnesene	18.74	96	429.41 ± 125.69	0.47 ± 0.13
α-humulene	18.87	CTA	3002.91 ± 320.52	3.28 ± 0.28
valencene	19.76	99	482.46 ± 93.57	0.53 ± 0.09
α-selinene	19.89	90	261.28 ± 107.87	0.28 ± 0.11
β-bisabolene	20.12	97	828.31 ± 201.79	0.90 ± 0.20
α-gurjunene	20.70	98	170.93 ± 32.38	0.19 ± 0.03
γ-selinene	20.80	96	1807.54 ± 413.43	1.97 ± 0.41
Selina-3,7(11)diene	20.99	99	2405.71 ± 311.73	2.63 ± 0.29
*Sesquiterpenoids*				
guaiol	22.29	96	587.34 ± 96.07	0.64 ± 0.11
γ-eudesmol	22.90	93	878.89 ± 97.01	0.96 ± 0.11
β-eudesmol	23.70	CTB	442.33 ± 76.32	0.49 ± 0.09
(+)-bulnesol	23.89	94	273.87 ± 43.55	0.30 ± 0.05
Total	-	-	91,324.74 ± 2774.36	-

^a^ GC-MS retention times (minutes). ^b^ Identification by means of standard mixture: Cannabis terpene A and B (CTA and CTB) or by means of GC-MS libraries with indication of qualifier (%). ^c^ S.D. = Standard deviation.

**Table 2:** Terpenes present in olive oil fractions obtained after inflorescences decarboxylation, expressed as mg/kg oil (mean ± standard deviation), shown in SPME/GC-MS analyses.

Compounds	Ret. Time (min) ^a^	Ident. (ST/Qual. %) ^b^	F1 (mg/kg, Mean ± S.D.) ^c^	F2 (mg/kg, Mean ± S.D.) ^c^	F3 (mg/kg, Mean ± S.D.) ^c^
*Monoterpenes*					
α-pinene	5.01	CTA	6120.28 ± 480.41	2547.44 ± 167.02	712.71 ± 56.82
Camphene	5.40	CTA	207.38 ± 17.6	62.94 ± 5.94	13.21 ± 2.30
β-pinene	6.08	CTA/B	1703.88 ± 217.10	422.61 ± 36.67	53.36 ± 4.18
β-myrcene	6.41	97	19,314.47 ± 830.54	6646.57 ± 481.01	285.35 ± 17.79
δ-3-carene	6.79	CTA/B	86.35 ± 20.35	48.72 ± 6.97	9.14 ± 0.93
limonene	7.40	CTA/B	6030.37 ± 103.55	991.04 ± 75.96	34.26 ± 9.21
γ-terpinene	8.17	CTA	92.97 ± 6.06	-	-
α-terpinolene	8.90	CTB	80.79 ± 19.84	-	-
*Monoterpenoids*					
l-fenchone	9.08	CTA	55.71 ± 21.62	-	-
Linalool	9.30	CTB	543.50 ± 79.71	-	-
d-α-Fenchyl alcohol	9.90	CTA	193.47 ± 24.52	92.18 ± 11.02	91.13 ± 7.16
Borneol L	11.43	CTB	34.93 ± 2.85	-	-
Terpinen-4-ol	11.67	90	17.48 ± 2.36	-	-
α-terpineol	12.10	CTB	80.50 ± 8.04	-	-
*Sesquiterpenes*					
*trans*-β-caryophyllene	17.90	CTB	2724.93 ± 615.49	-	-
α-bergamotene	18.20	91	31.76 ± 5.56	-	-
*trans*-β-farnesene	18.75	96	11.69 ± 9.70	8.09 ± 1.11	9.61 ± 0.54
α-humulene	18.87	CTA	59.93 ± 19.79	-	-
α-selinene	19.89	92	6.54 ± 4.80	-	-
β-bisabolene	20.00	91	16.56 ± 12.39	-	-
Selina-3,7(11)diene	20.99	96	14.04 ± 12.37	-	-
Total	-	-	37,390.38 ± 723.16	10,819.59 ± 187.83	1208.78 ± 31.12

^a^ GC-MS retention times (minutes). ^b^ Identification by means of standard mixture: *Cannabis* terpene A and B (CTA and CTB) or by means of GC-MS libraries with indication of qualifier (%). ^c^ S.D. = Standard deviation.

**Table 3:** Terpenes present in oils obtained after mixing and diluting fractions 1÷3 (Mix F1 ÷ 3 dil.) and after extraction at 60 and 100 °C (Ext. 60 and Ext. 100), expressed as mg/kg oil (mean ± standard deviation), with the increase expressed as a percentage compared to the oil sample before extraction.

Compounds	Mix F1 ÷ 3 dil. (mg/kg, Mean ± S.D.) ^a^	Ext. 60 (mg/kg, Mean ± S.D.) ^a^	Ext. 100 (mg/kg, Mean ± S.D.) ^a^	Increase % Ext. 60 ^b^	Increase % Ext. 100 ^b^
*Monoterpenes*					
α-pinene	639.89 ± 11.59	1001.30 ± 165.37	1185.22 ± 114.54	56.48	85.22
Camphene	19.30 ± 0.15	32.63 ± 5.53	38.30 ± 4.52	69.05	98.40
β-pinene	173.96 ± 2.63	287.73 ± 35.62	329.46 ± 26.45	65.40	89.38
β-myrcene	3866.84 ± 112.21	5963.38 ± 268.21	6422.52 ± 419.28	54.22	66.09
δ-3-carene	27.61 ± 8.05	31.68 ± 7.46	38.31 ± 4.57	14.73	38.76
limonene	906.52 ± 63.54	1559.04 ± 145.29	1720.05 ± 104.28	71.98	89.74
γ-terpinene	8.49 ± 1.92	23.49 ± 3.48	28.53 ± 2.82	176.63	235.88
α-terpinolene	−	14.41 ± 1.78	18.11 ± 5.20	+	+
*Monoterpenoids*					
l-fenchone	−	16.59 ± 3.88	21.12 ± 3.93	+	+
Linalool	16.86 ± 2.41	207.45 ± 52.45	216.71 ± 38.32	1130.42	1185.33
d-α-Fenchyl alcohol	68.45 ± 10.65	121.70 ± 16.08	119.17 ± 18.53	77.79	74.10
Borneol L	−	24.51 ± 5.94	26.79 ± 5.74	+	+
Terpinen-4-ol	−	7.87 ± 2.84	8.82 ± 2.93	+	+
α-terpineol	−	71.44 ± 23.03	72.94 ± 17.60	+	+
*Sesquiterpenes*					
*trans*-β-caryophyllene	−	235.29 ± 80.56	237.78 ± 63.10	+	+
α-bergamotene	−	19.94 ± 8.60	19.07 ± 6.91	+	+
*trans*-β-farnesene	6.04 ± 1.32	9.65 ± 2.34	8.43 ± 4.77	59.64	39.45
α-humulene	−	62.57 ± 25.17	63.80 ± 17.63	+	+
α-selinene	−	−	4.36 ± 3.10	+	+
β-bisabolene	−	21.00 ± 6.84	28.93 ± 12.66	+	+
Selina-3,7(11)diene	−	36.68 ± 11.80	32.59 ± 8.26	+	+
*Sesquiterpenoids*					
guaiol	−	9.92 ± 5.31	8.67 ± 2.89	+	+
γ-eudesmol	−	17.30 ± 7.35	16.23 ± 4.69	+	+
β-eudesmol	−	5.78 ± 3.38	6.50 ± 3.14	+	+
Total	5733.97 ± 167.35 ^A^	9775.56 ± 678.04 ^B^	10,672.39 ± 723.05 ^B^	70.48	86.13

^a^ S.D. = Standard deviation. The − sign indicates that the corresponding compound was not present. ^b^ The sign + indicates that the corresponding compound is present in the Ext. 60 and Ext. 100, but not in the starting oil sample (Mix F1 ÷ 3 dil.); therefore, the Increase % could not be calculated. ^A,B^ Tukey’s post hoc test groups.

**Table 4:** Terpene recovery from inflorescences after decarboxylation (F1, F2, F3, Mix F1 ÷ 3 dil.) and after MAE at 60 and 100 °C (Ext. 60 and Ext. 100), expressed as percentage (mean ± standard deviation), obtained from semiquantitative SPME/GC-MS analyses.

Samples ^a^	Total Terpenes (mg/kg)	Total Terpenes (mg in the Sample)	Terpene Recovery (*w*/*w* %) ^b^
Inflorescences (100 g)	91,324.74 ± 2774.36	9132.47	-
F1 (100 mL olive oil)	37,390.38 ± 723.16	3439.92	37.67 ± 0.73
F2 (100 mL olive oil)	10,819.59 ± 187.80	995.40	10.90 ± 0.19
F3 (100 mL olive oil)	1208.78 ± 31.10	111.21	1.22 ± 0.03
Mix F1 ÷ 3 dil. (260 mL of 1:1:1 mixture of F1, 2 and 3/1000 mL olive oil)	5733.97 ± 167.35	5275.25	57.76 ± 1.69
Ext 60 (500 mL Mix F1 ÷ 3 dil./ 50 g inflorescences)	9775.56 ± 678.04	4496.76	98.48 ± 6.83
Ext 100 (500 mL Mix F1 ÷ 3 dil./ 50 g inflorescences)	10,672.39 ± 723.05	4909.30	107.51 ± 7.28

^a^ An olive-oil density of 0.92 g/mL was considered for calculations. ^b^ Recovery % was calculated on the terpene amount present in the initial amount of inflorescences.

## Data Availability

All the available data have been reported in the manuscript.
